# Elevated MicroRNA-31 Expression Regulates Colorectal Cancer Progression by Repressing Its Target Gene SATB2

**DOI:** 10.1371/journal.pone.0085353

**Published:** 2013-12-30

**Authors:** Min-Hui Yang, Jiang Yu, Na Chen, Xiao-Yan Wang, Xiang-Yu Liu, Shuang Wang, Yan-Qing Ding

**Affiliations:** 1 Department of Pathology, Nanfang Hospital, Southern Medical University, Guangzhou, China; 2 Department of Pathology, School of Basic Medical Sciences, Southern Medical University, Guangzhou, China; 3 Department of General Surgery, Nanfang Hospital, Southern Medical University, Guangzhou, China; 4 Health Science Center, Peking University, Beijing, China; Wayne State University School of Medicine, United States of America

## Abstract

Several studies have brought about increasing evidence to support the hypothesis that miRNAs play a pivotal role in multiple processes of carcinogenesis, including cell growth, apoptosis, differentiation, and metastasis. In this study, we investigated the potential role of miR-31 in colorectal cancer (CRC) aggressiveness and its underlying mechanisms. We found that miR-31 increased in CRC cells originated from metastatic foci and human primary CRC tissues with lymph node metastases. Furthermore, the high-level expression of miR-31 was significantly associated with a more aggressive and poor prognostic phenotype of patients with CRC (*p* < 0.05). The stable over-expression of miR-31 in CRC cells was sufficient to promote cell proliferation, invasion, and migration *in vitro*. It facilitated tumor growth and metastasis *in vivo* too. Further studies showed that miR-31 can directly bind to the 3’untranslated region (3’UTR) of SATB2 mRNA and subsequently repress both the mRNA and protein expressions of SATB2. Ectopic expression of SATB2 by transiently transfected with pCAG-SATB2 vector encoding the entire SATB2 coding sequence could reverse the effects of miR-31 on CRC tumorigenesis and progression. In addition, ectopic over-expression of miR-31 in CRC cells induced epithelial-mesenchymal transition (EMT). Our results illustrated that the up-regulation of miR-31 played an important role in CRC cell proliferation, invasion, and metastasis *in vitro* and *in vivo* through direct repressing SATB2, suggesting a potential application of miR-31 in prognosis prediction and therapeutic application in CRC.

## Introduction

Colorectal cancer (CRC) is one of the most common cancers in the world. Although several kinds of treatments have been developed recently for the patients with CRC, poor prognosis continues to be in patients with advanced CRC[1]. Most CRC deaths have been associated with tumor invasion and metastasis. So, understanding the underlying molecular mechanisms of CRC metastasis is of crucial significance in developing therapeutic strategies for advanced CRC patients. 

microRNAs (miRNAs) are an abundant class of highly conserved, short, regulatory (about 22 nt) non-coding RNAs that are widely expressed in living organisms. They bind to the 3’UTR of mRNA, causing either mRNA molecule degradation or translational inhibition[2]. miRNAs have diverse functions, including the regulation of cellular differentiation, proliferation, and apoptosis[3,4]. Therefore, quite a few studies have reported the pivotal role of miRNAs in the multiple processes of carcinogenesis, including metastasis[3,5,6]. Moreover, expression analyses have revealed characteristic miRNA signatures in specific human cancers[7–9]. 

Several investigators reported that miR-31 up-regulated in CRC[10–12] and squamous cell carcinoma of tongue[13], but down-regulated in breast cancer[14], gastric cancer[15], malignant mesothelioma[16] and pancreatic cancer[17] using qRT-PCR. But, the clinical prognostic significance, function and regulatory activity of miR-31 in CRC have not been completely understood yet. In this study, we explored the unambiguous role of miR-31 in CRC and found that the up-regulation of miR-31 was associated with the aggressive phenotypes of CRC and poor prognosis in patients. Further investigations revealed that the over-expression of miR-31 in CRC led to increase tumor cell proliferation and motility *in vitro* and *in vivo*. The present study determines a positive role of miR-31 in carcinogenesis, invasion, and migration, via repressing SATB2 expression in CRC. 

## Materials and Methods

### Ethics statement

The use of tissues for this study has been approved by the ethics committee of Nanfang Hospital, Southern Medical University. All of the patients signed the informed consent before use of these clinical materials for research purposes. This study was carried out in strict accordance with the recommendations in the Guide for the Care and Use of Laboratory Animals of the National Institutes of Health. The protocol was approved by the Committee on the Ethics of Animal Experiments of Southern Medical University (Permit Number: SYXK2011–0074). All surgery was performed under sodium pentobarbital anesthesia, and all efforts were made to minimize suffering.

### Tissue preparation and cell culture

Fresh and formalin-fixed, paraffin-embedded, colorectal tumor tissue samples were obtained from patients with a diagnosis of primary CRC and then underwent elective surgery in Nanfang Hospital, Southern Medical University (Guangzhou, China). The use of tissues for this study has been approved by the ethics committee of Nanfang Hospital, Southern Medical University. A total of 31 cases of fresh CRC tissue were freshly frozen in liquid nitrogen and stored at -80°C until further use. And 143 cases of archived CRC tissue samples were collected and used in clinicopathological and prognostic investigation of miR-31. No patient received any pre-operative chemotherapy or radiotherapy. A comprehensive set of clinicopathological data were possessed, including age, gender, size of primary tumor, tumor differentiation, T stage, lymph node metastasis and distant metastasis. Complete follow-up, ranging from 1-96 months, was available for the cohort of 143 patients, and the median survival was 56 months.

The human embryonic kidney cells 293T and the human CRC cell lines DLD-1, HCT116, SW480, SW620, Lovo were obtained from a cell bank at the Chinese Academy of Sciences (Shanghai, China). In previous studies, we have described a subclone named M5 with enhanced metastatic abilities in liver. We have also described a subclone named SCP 51 with high metastatic abilities in liver and lymph node. These subclones were isolated by *in vivo* selection of SW480 cells through a process described in previous studies[18–20]. All CRC cell lines were cultured in RPMI 1640 medium (Gibco, Gaithersburg, MD, USA) with 10% fetal bovine serum (HyClone, Logan, USA) and 100 U/ml penicillin / streptomycin (Gibco). They were maintained in a humidified chamber with 5% CO_2_ at 37°C. 293T was maintained in Dulbecco’s modified Eagle’s medium (DMEM) supplemented with 10% FBS.

### RNA isolation and quantitative real-time PCR

Total RNA was extracted with TRIzol Reagent (Invitrogen, Carlsbad, CA). cDNA was synthesized with the PrimeScript RT reagent Kit (Promega, Madison, WI, USA). A stem-loop quantitative RT-PCR was carried out to detect expression of mature miR-31 with the ABI TaqMan^®^  MicroRNA Assay kit (Applied Biosystems, Foster City, USA) and gene-specific primers (Applied Biosystems, Foster City, USA) using an ABI 7500 Real-Time PCR system. The assay was performed in triplicate for each case to allow for assessment of technical variability.

### In situ hybridization and evaluation of staining of miR-31

In situ hybridization (ISH) was performed according to the manufacturer’s protocol (Exiqon, Vedbaek, Denmark). Paraffin-embedded sections (4 μm thick) were deparaffinized with xylene and rehydrated with dilute ethanol of reagent grade. The slides were treated with proteinase K at 37°C for 20 minutes. Then, they were prehybridized in a hybridization solution at 50°C for 2 hours. Subsequently, 40 nM of a locked nucleic acid-modified, 5’ digoxigenin (DIG)-labeled oligonucleotide probe of hsa-miR-31 or a scrambled control probe (Exiqon) was added to the hybridization solution and hybridized at a temperature of 50°C overnight. An alkaline phosphate conjugated anti-DIG antibody (Roche, Mannheim, Germany) was applied. After being washed in staining solution, the sections were incubated in NBT/ BCIP developing solution (Roche) at 37°C for 15 to 30 minutes. Then, they were counterstained with nuclear fast red. 

To assess the patients’ clinical characteristics, the ISH stained tissue sections were reviewed and scored separately by two blinded pathologists. Staining for miR-31 was assessed using a relatively simple, reproducible scoring method[21,22]. On a scale of 0 to 3, the staining intensity was scored as follows: negative (no staining, 0), weak (light blue, 1), medium (blue, 2), or strong (dark blue, 3). The extent of the staining is defined as the percentage of positive staining areas of tumor cells or normal colonic epithelial cells in relation to the whole tumor area or entire section for the normal samples. The extent of staining was scored on a scale of 0 to 4 as follows: 0, 0%; 1, 1–25%; 2, 26–50%; 3, 51–75%; and 4, 76–100%. The sum of the staining-intensity and staining-extent scores was used as the final staining score for miR-31 (0–7). For statistical analysis, a final staining score of ≥ 3 was considered to be high.

### Vector preparation

A 184-bp DNA fragment corresponding to pre-miR-31 was selected, and the flanking sequence was amplified and cloned into pLVTHM lentiviral vector (http://www.addgene.org/Didier_Trono) to generate pLV-miR-31 expression vector. pLVTHM lentiviral vector encodes enhanced green fluorescent protein (EGFP) that has been optimized for brighter fluorescence and greater expression in mammalian cells. The previously described vector pCAG-SATB2[19] was used to up-regulate SATB2 expression. 

The full-length 3'UTR of SATB2 (2711bp) was cloned and then inserted into the downstream of luciferase reporter gene of pGL3-control Vector (Promega, Madison, WI, USA). This was done to generate pGL3-SATB2-3’UTR. Two putative miR-31 binding sites at 3’UTR of SATB2 were site-directed and mutated, respectively using GeneTailor Site-Directed Mutagenesis System (Invitrogen). All plasmids were verified by sequencing. All primer sequences for detection and miRNA sequences are provided in Table S1. 

### Lentivirus production and infection

Virus particles were harvested 48 hours after pLV-miR-31 transfection with the envelope plasmid pMD2.G and the packaging vector psPAX2 into 293T cells using lipofectamine 2000 reagent (Invitrogen). CRC cells were infected with the recombinant lentivirus-transducing units plus 8 mg/ml Polybrene (Sigma, St Louis, Missouri, USA) and then subjected to FACS analysis for GFP expression to gain CRC cells with stable over-expression of miR-31. The empty lentiviral vector pLVTHM was used as the control (miR-con).

### Oligonucleotide transfection

miR-31 mimics and antisense inhibitors containing 2’-OMe (*O*-methyl) modifications were synthesized by GenePharma (Shanghai, China). Oligonucleotide transfection was performed with Lipofectamine 2000 reagent (Invitrogen). 

### Luciferase reporter assay

In the presence of either miR-31 or miR-con, the firefly luciferase construct was co-transfected with a control *Renilla* luciferase vector pRL-CMV (Promega) into SW480 cells. A dual luciferase assay (Promega) was performed 48 hours after transfection. The experiments were performed in triplicate independently.

### Cell proliferation assay and cell-cycle analysis

Cells were seeded in 96-well plates at 2×10^3^ per well. Cell proliferation was evaluated using Cell Counting Kit-8 (CCK-8, Dojindo, Rockville, USA) according to the manufacturer's instructions. For cell-cycle analysis, cells were plated in 6-well plates at 5×10^5^ per well. The cell-cycle distribution was analyzed by propidium iodide (Sigma-Aldrich) staining and flow cytometry[23]. 

### Colony formation assay

The cells were plated in 6-well plates at 2×10^2^ per well and maintained in RPMI1640 containing 10% FBS for 2 weeks. After 2 weeks, the cells were washed twice with PBS, fixed with methanol and stained with 0.5% crystal violet. The number of colonies was counted under a microscope[24]. 

### Wound healing and invasion assays

Cell migration was assessed by measuring the movement of cells into a scraped, acellular area created by a 200 μL pipette tube, and the spread of wound closure was observed after 0 and 48 hours, respectively. Photographs were taken to assess the level of migration in each group of transfected cells. Migration was quantified by counting the total number of cells that migrated toward the original wound field. For invasion assay, matrigel-coated chambers (BD Biosciences, San José, CA, USA) containing 8 µm pores were used. Cells were seeded into the upper chambers (coated in matrigel) at 2×10^5^ concentration in serum-free medium. The lower chamber of the transwell was filled with culture media containing 10% FBS as a chemo-attractant. After the chambers were incubated at 37 °C for 48 hours, non-invaded cells on the top of the transwell were scraped off with a cotton swab. Successfully translocated cells were fixed with 10% formalin. Then, they were stained with 0.1% crystal violet for 30 minutes and counted under a light microscope.

### *In vivo* functions assays

Balb/C-nu/nu athymic nude mice (4-6 weeks old) were purchased from the Laboratory Animal Centre of Southern Medical University. Mice were housed under pathogen free conditions in a 12 hours dark/light cycle and *ad libitum* access to food and filtered water. Animals used for experimentation received humane care. For tumor growth assay, a total of 2 × 10^6^ cells of SW480 with stable over-expression miR-31, or control cells, were injected subcutaneously in left and right flank of mice (n = 6 per group). Then, fluorescence emitted by cells was collected and images were analyzed through a whole-body GFP imaging system (Lighttools, Encinitas, CA) that could visualize real-time tumor growth. Tumor size was measured using digital calipers every three days. After 30 days of monitoring, mice were sacrificed by cervical dislocation and tumors were dissected. Tumor volume was calculated as follows: Volume = (D × d^2^)/2, where D meant the longest diameter and d meant the shortest diameter.

 For establishing metastatic model, mouse cecum was exteriorized by laparotomy under sodium pentobarbital anesthesia (6mg/100g body weight). The subcutaneous tumors were diced into 1 mm^3^ cubes and implanted into the mesentery at the cecum terminus of mice. Then, the gut was returned to the abdominal cavity and closed with surgical drapes. Heating pads were used to help postoperative recovery for mice after xenografts implantation. Animal health status was monitored after surgery daily, including their physical activity, hydration, eating and drinking habits and ambulation. Body weight of control and SW480/miR-31 mice was also recorded every three days. Humane endpoints had been planned at the end of experiment and as a means to relive pain or distress. An integrated criterion for need to euthanasia was carried out in our study, including physical deficit, sickness, distress, immobility, weight loss and maximum tumor size. When they appeared evident physical deficit, such as dry or dull eyes, tacky mucous membranes, hunched, ambulation reduction, disregarded observer, dyspnea or cachexia, mice were deemed to meet the criteria of euthanasia and were sacrificed to minimize suffering and distress. At the end of the experiment (8 weeks), the still alive mice were sacrificed by cervical dislocation. The organs were removed and fixed with 10% neutral buffered formalin. Subsequently, consecutive tissue sections were obtained and stained with haematoxylin-eosin (H&E) to observe the metastatic nodules of organs under microscope. All experimental procedures were performed in strict accordance with the recommendations in the Guide for the Care and Use of Laboratory Animals of the National Institutes of Health. The protocol was approved by the Committee on the Ethics of Animal Experiments of Southern Medical University. All necessary steps were taken to minimize suffering and distress to the mice. 

### Western blot analysis

Protein lysates were separated by 10% SDS-PAGE gel electrophoresis and transferred to PVDF membrane (Amersham Pharmacia Biotech, NJ, USA). The membrane was probed with the following antibodies: anti-SATB2 (Abcam, Cambridge, UK), anti-E-cadherin (Cell Signaling Technology, Inc), anti-N-cadherin (Cell Signaling Technology), anti-Vimentin (Cell Signaling Technology), and anti-β-catenin (Cell Signaling Technology). Finally, the membrane was probed with HRP (horseradish peroxidase)-labeled goat-antimouse IgG (Santa Cruz Biotechnology, USA) and detected by chemiluminescence. A polyclonal anti-β-actin antibody (Santa Cruz Biotechnology) was used as a protein-loading control. The intensity of protein fragments was quantified with the Quantity One software (4.5.0 basic, Bio-Rad).

### Immunohistochemistry (IHC)

The tissue blocks were cut into 4 μm sections and processed for IHC in accordance with a previously described protocol[19]. 

### Statistical analysis

All statistical analyses were performed using the SPSS 16.0 statistical software package. In at least three independent experiments, data were presented in terms of mean ± SEM. Differences between variables were assessed by the following three statistical tests: χ^2^ test, Fisher’s exact test, or One-way ANOVA. For patients with different levels of miR-31 expression, survival curves were plotted using the Kaplan–Meier method and compared using the log-rank test. Multivariate survival analysis was performed on all parameters that were found to be significant in univariate analysis using the Cox regression model. A *p* value less than 0.05 was considered as statistical significance.

## Results

### miR-31 was up-regulated in metastatic CRC cells and human primary CRC tissues with lymph node metastases

**Figure 1:**
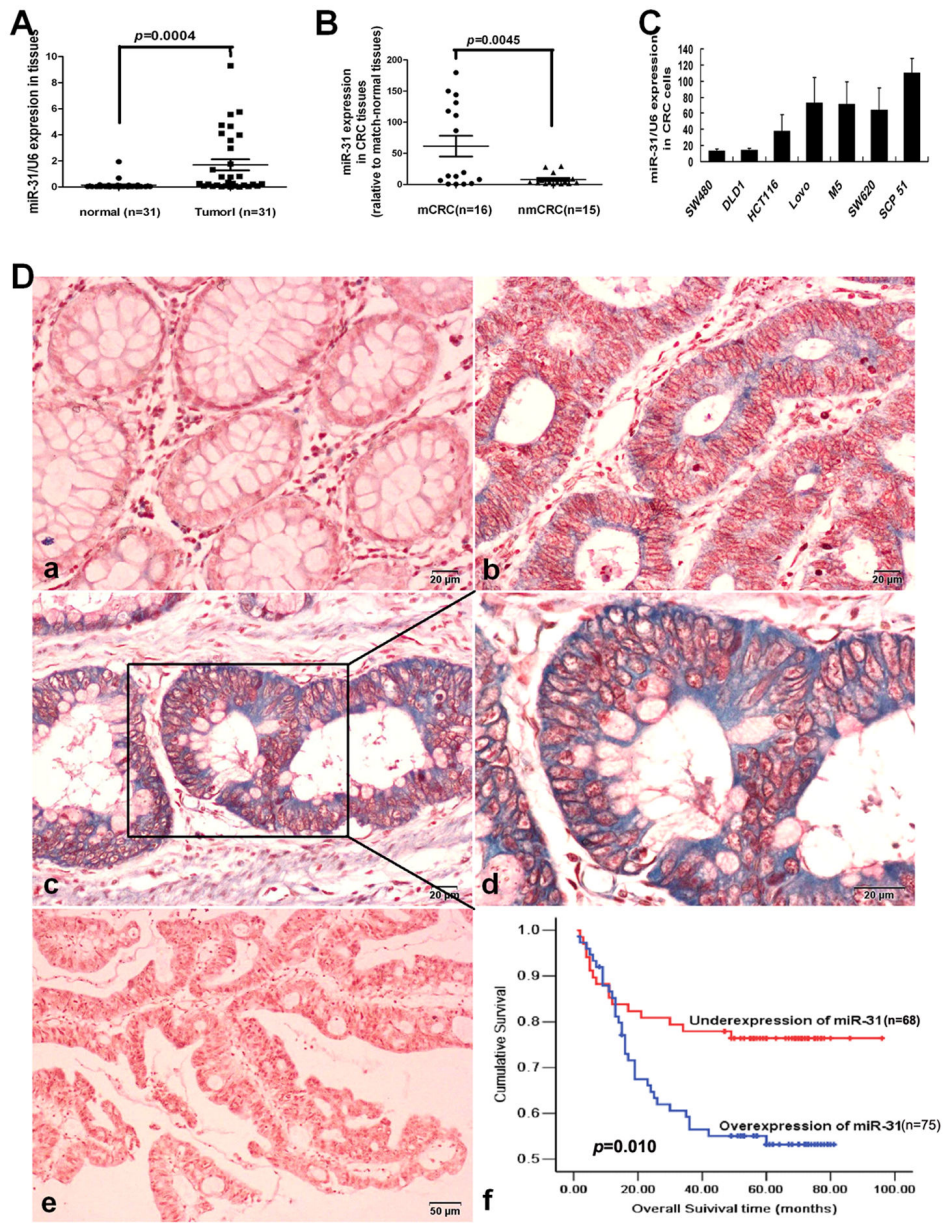
The levels of miR-31 expression in CRC cells and tissues by qRT-PCR or *in* *situ* hybridization and its prognostic value in patients with CRC. (A) Expression levels of mature miR-31 in paired CRC and adjacent normal tissues. (B) The miR-31 expression in CRC tissues with or without metastases relative to match-normal tissues. nmCRC denotes CRC tissues without metastases; mCRC denotes CRC tissues with lymph node metastases. (C) Expression of miR-31 in CRC cell lines and subclones. miRNA abundance was normalized to U6 RNA. (D) Expression analysis of miR-31 in normal colorectal mucosa and CRC tissues by *in* *situ* hybridization. (a) Negative expression of miR-31 in normal colorectal mucosa; (b) Low expression of miR-31 in CRC tissues; (c, d) High expression of miR-31 in CRC tissues; (e) negative control; (f) Kaplan-Meier analysis for survival of patients with CRC according to miR-31 expression. Log Rank = 6.682, *p* = 0.010.

We first examined miR-31 expression by a stem-loop quantitative RT-PCR in a panel of CRC cell lines and 31 pairs of CRC and adjacent non-neoplastic mucosa tissues. The results showed that miR-31 was remarkably up-regulated in CRC tissues compared with adjacent non-neoplastic normal tissues (*p*=0.0004, Figure 1A). It was observed that the up-regulation of miR-31 in tumor samples was associated with lymph-node metastasis to a significant extent (*p*=0.0045, Figure 1B). In patients with lymph node metastases, the relative mean expression of miR-31 was over 7.82 fold higher than that in patients without metastases (61.54±16.68 *vs*.7.87±2.47). In addition, miR-31 was low in SW480 and DLD-1 cells, which originate from primary tumors, whereas it was relatively high in SW620, Lovo, M5 and SCP51 cells, which originate from metastatic foci (Figure 1C), especially in M5 and SCP51 cell lines, which have high metastatic potential among the CRC cell lines. This association indicated that miR-31 might well have a pivotal role in CRC metastasis.

### Over-expression of miR-31 was associated with an aggressive phenotype and poor prognosis of patients with CRC

To further investigate the clinicopathological and prognostic significance of miR-31 levels, we measured miR-31 expression in a large cohort of 143 archived paraffin-embedded CRC and normal colon tissues using ISH. We observed that 75/143 (52.45%) CRCs had high-level expression miR-31, whereas 11/55 (20%) normal mucosa tissues had high-level expression of miR-31 (*p*<0.001). The correlation analysis between clinicopathological characteristics and miR-31 level showed high-level expression of miR-31 was significantly associated with T-stage (*p*=0.045), lymph node metastasis (*p*=0.001), and distant metastasis (*p*=0.026) in patients with CRC, however, not associated with age, sex, tumor site, tumor size, and tumor differentiation degree (*p*>0.05, Table 1) .

**Table 1:** Correlation between the clinicopathological features and expression of miR-31.

**Characteristics**	**n**	**miR-31 expression**		
		**low (%)**	**high (%)**	***P* value**
**Gender**				
**Male**	**87**	**39(44.83)**	**48(55.17)**	**0.416**
**Female**	**56**	**29(51.79)**	**27(48.21)**	
**Age(years)**				
**<50**	**65**	**35(53.85)**	**30(46.15)**	**0.169**
**≥50**	**78**	**33(42.31)**	**45(57.69)**	
**Tumor site**				
**Proximal colon**	**42**	**20(47.62)**	**22(52.38)**	**0.369**
**Distal colon**	**37**	**21(56.76)**	**16(43.24)**	
**Rectum**	**64**	**27(42.19)**	**37(57.81)**	
**Tumor size(cm in diameter)**				
**<5**	**45**	**19(42.22)**	**26(57.78)**	**0.387**
**≥5**	**98**	**49(50.00)**	**49(50.00)**	
**Tumor differentiation**				
**Good**	**13**	**6(46.15)**	**7(53.85)**	**0.979**
**Moderate**	**104**	**50(48.08)**	**54(51.92)**	
**Poor**	**26**	**12(46.15)**	**14(53.85)**	
**T-stage**				
**1-2**	**23**	**16(69.57)**	**7(30.43)**	**0.045**
**3**	**87**	**40(45.98)**	**47(54.02)**	
**4**	**33**	**12(36.37)**	**21(63.63)**	
**N-stage**				
**0**	**59**	**38(64.41)**	**21(35.59)**	**0.001**
**1-2**	**84**	**30(35.71)**	**54(64.29)**	
**Distant metastasis**				
**0**	**94**	**51(54.26)**	**43(45.74)**	**0.026**
**1**	**49**	**17(34.69)**	**32(65.31)**	

To evaluate the prognostic value of miR-31 for CRCs, we analyzed the association between miR-31 expression and survival duration using Kaplan-Meier analysis with the log-rank test. The results revealed that high-level expression of miR-31 was correlated with short survival time of patients with CRC (51.85±3.78 *vs*.76.64±4.30, *p*=0.010, Figure 1D). Furthermore, multivariate Cox regression analysis indicated that high-level expression of miR-31 is an independent prognostic factor for poor survival of patients with CRC (Table 2).

**Table 2:** Overall survival analyses by univariate and multivariate COX regression analysis.

**Variables**	**Univariate analysis**			**Multivariate analysis**		
	***P* value**	**HR**	**95% Confidence interval**	***P* value**	**HR**	**95% Confidence interval**
**Gender**	0.516	0.826	0.464-1.471			
**Age**	0.391	0.785	0.451-1.366			
**Tumor site**	0.915	0.982	0.707-1.365			
**Tumor size**	0.581	1.190	0.642-2.207			
**Tumor differentiation**	0.074	1.648	0.953-2.850			
**T-stage**	0.001	2.262	1.419-3.606	0.032	1.695	1.045-2.750
**N-stage**	0.041	1.563	0.846-2.728	0.604	0.847	0.453-1.586
**M-stage**	<0.001	4.338	2.441-7.707	<0.001	3.563	1.940-6.545
**miR-31 expression**	0.012	2.145	1.182-3.890	0.048	1.877	1.007-3.488

### Exogenetic over-expression of miR-31 promoted CRC cells growth, invasion, and migration *in vitro*

**Figure 2:**
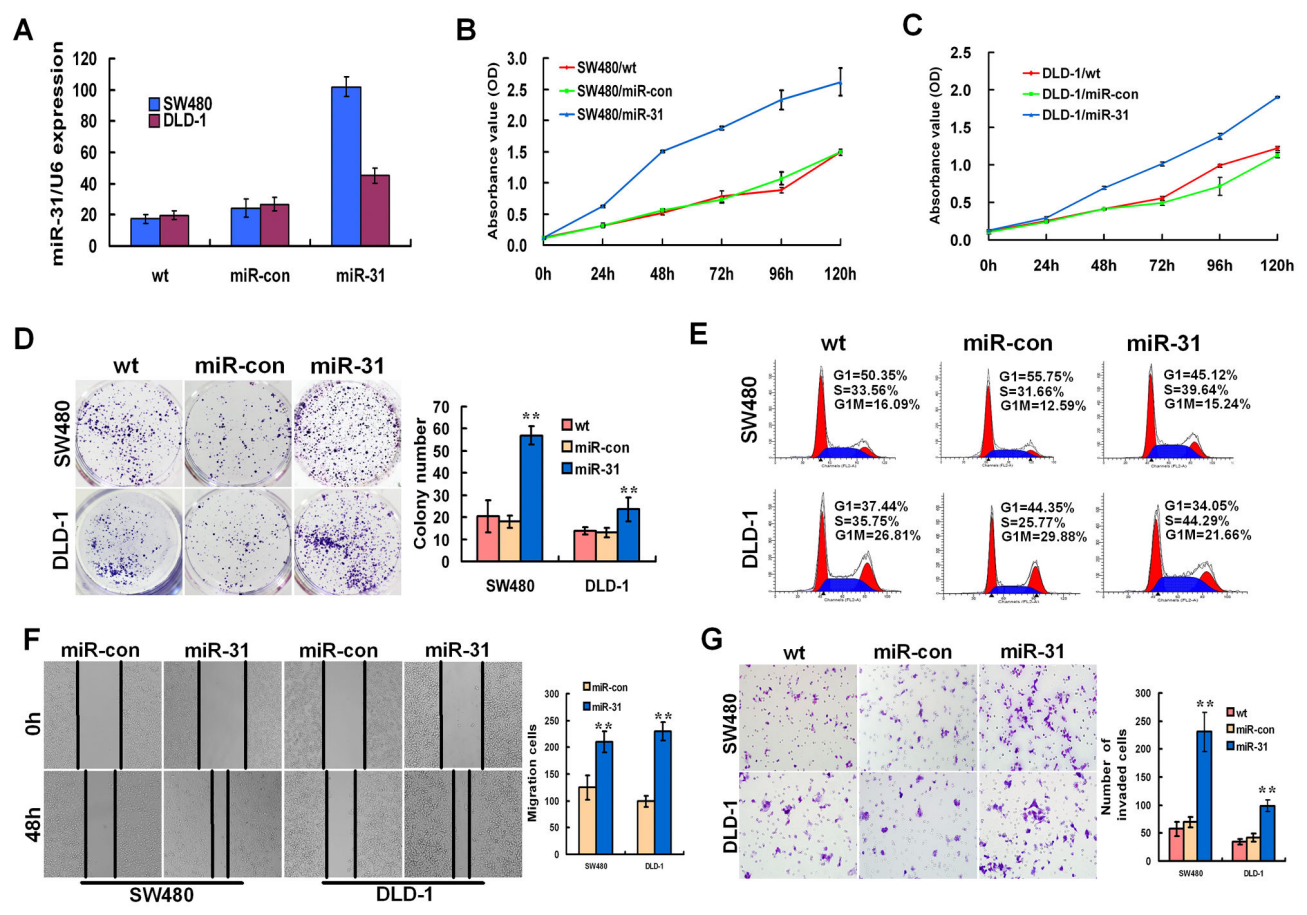
miR-31 promoted aggressive phenotypes of CRC cells *in* *vitro*. (A) Real-time RT-PCR analysis of miR-31 expression level in SW480 and DLD-1 cells after ectopic over-expression of miR-31. (B, C) Effect of miR-31 on cell proliferation was measured by CCK-8 assay. (D) Comparison of colony formation effect of CRC cell lines. (E) Cell-cycle distribution of CRC cells infected with miR-31 was detected by flow cytometry analysis. (F, G) Effects of miR-31 ectopic over-expression on cell motilities and invasiveness were determined using wound-healing (F) and matrigel invasion (G) assays. Data were presented as mean ±SD. The results were reproducible in three independent experiments. * *p* < 0.05, ** *p* <0.001.

To explore the potential biological function of miR-31 in CRC tumorigenesis and progression, SW480 and DLD-1 cells were infected with pLV-miR-31 or pLV-con, respectively. This was done to establish two stable miR-31-overexpression cell lines and two mock cell lines (miR-con). An increased expression of miR-31 upon infection in two cell lines was confirmed by real-time RT-PCR (Figure 2A). As shown in Figure 2B-2D, the over-expression of miR-31 increased cancer cell proliferation and its ability to form colonies than that in the mock cells and wild type non-infected cells (*p*<0.001). Flow cytometry and cell cycle analysis revealed a significant decrease in the percentage of cells in the G1/G0 phase (*p*=0.004 in SW480*, p*=0.008 in DLD1) and an increase in S phase of CRC cells treated with miR-31 (*p*=0.002 in SW480*, p*=0.001 in DLD1, Figure 2E). 

After observing the miR-31-mediated growth aggrandizement, the effect of miR-31 on invasive capacity of CRC cells was characterized by the wound-healing and matrigel invasion assay. The results indicated that exogenetic expression of miR-31 in CRC cells caused a significant increase in cell migration using a wound-healing assay (*p*=0.001 in SW480*, p*<0.001 in DLD1, Figure 2F). Matrigel invasion assay also illustrated that the over-expression of miR-31 markedly induced invasiveness of CRC cells (*p*<0.001 in both SW480 and DLD1, Figure 2G). These results indicated that over-expression of miR-31 was sufficient to promote both cell proliferation and migration *in vitro*. 

### miR-31 facilitated CRC cells tumor growth and metastasis *in vivo*

**Figure 3:**
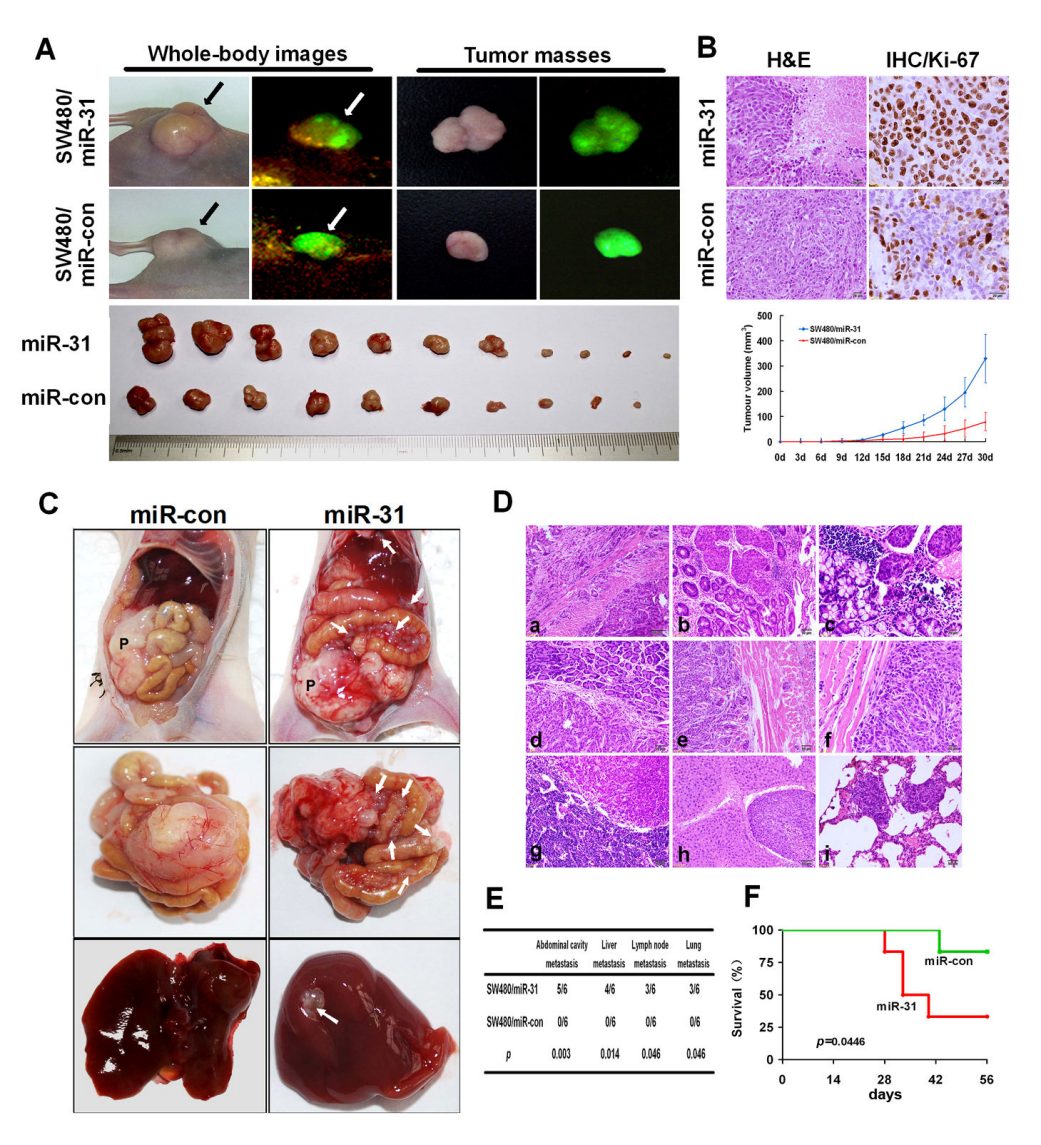
miR-31 enhanced tumor growth, invasion and metastasis *in* *vivo*. (A) External whole-body fluorescence images of SW480/miR-31 and SW480/miR-con mice and tumors (upper) and images of subcutaneous tumor of mice (lower) were obtained. Arrows indicate subcutaneous cancers. (B) Representative photographs of H&E and immunohistochemical staining for Ki-67 antibody of primary cancer tissues (upper) and growth curve of tumor volumes (lower). Each data point represents the mean ±SD. (C) The whole-body images of metastasis in mice. P denotes primary tumor. White arrows point to the metastatic nodules. (E) Histological images of metastatic nodules in organs. Xenograft tumors with miR-31-overexpression significantly formed the invasion of caecal wall (a,b), seeding metastasis of colon (c) , stomach wall (d), abdominal wall (e) and diaphragm (f), and metastasis of lymph node (g), liver (h) and lung (i). (E) Incidence of metastasis in mice that implanted with SW480/ miR-31 or miR-con cells. (F) Survival analysis of SW480/ miR-31 and SW480/miR-con mice.

Since miR-31 promotes growth, migration, and invasion of CRC cells *in vitro*, we tempted to determine whether miR-31 could facilitate tumor growth and metastasis *in vivo*. For this purpose, SW480 cells with stable over-expression miR-31 (SW480/miR-31) and control cells (SW480/miR-con) were subcutaneously inoculated in nude mice. As shown in Figure 3A, the speed of tumor growth in the SW480/miR-31 group was significant than that of the SW480/miR-con group (*p*<0.05, Figure 3A and 3B). IHC staining showed that the tumors of control group displayed much lower Ki-67 index than that of miR-31 over-expression group (Figure 3B).

To mimic human CRC and evaluate the *in vivo* effect of miR-31 on metastasis of CRC, SW480/miR-31 and SW480/miR-con cells were orthotropically implanted into the caecum terminus of individuals. Between 4 and 8 weeks after xenografts implantation, 4/6 experimental mice and 1/6 control mice had evident physical deficit because of increased tumor burden. Since meeting the criterion of humane endpoint, these mice were sacrificed to relieve suffering and distress. The remaining alive mice were sacrificed at the end of the experiment (8 weeks). The animal experiment results showed that miR-31-overexpression SW480 cells exhibited dramatic metastasis of liver and peritoneal cavity, whereas SW480/miR-con cells only caused tumor increases without any metastasis (Figure 3C). H&E staining clearly showed invasion in intestinal wall and metastasis of stomach, diaphragm, abdominal wall, liver, lymph node, and lung in SW480/miR-31cells groups (*p*<0.05,Figure 3D and 3E). In addition, it was revealed that miR-31 over-expression led to shorter overall survival times of the animals (*p*<0.05, Figure 3F).

### Inhibition of miR-31 reduced the growth, invasion, and migration of CRC cells *in vitro*

**Figure 4:**
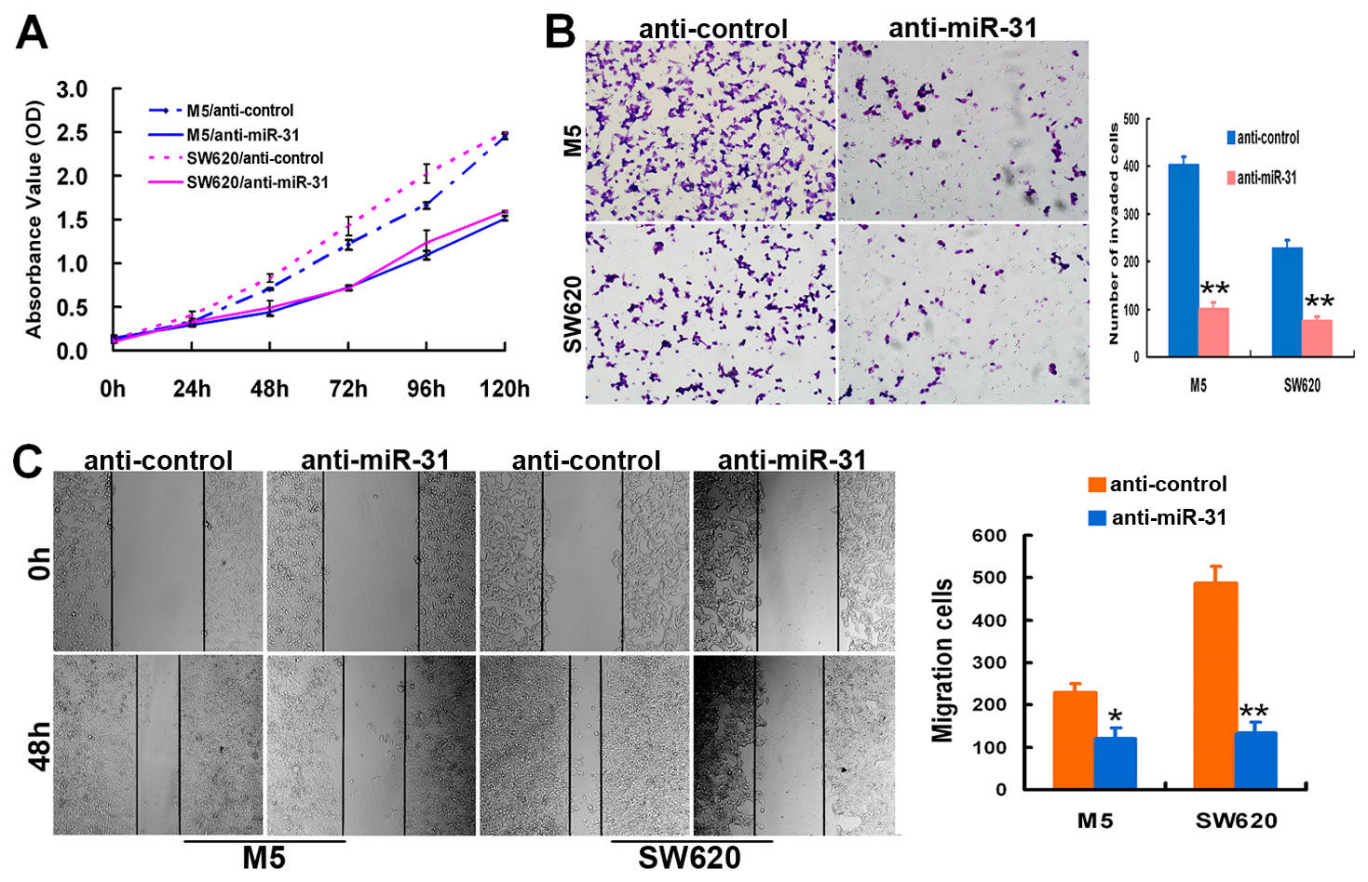
The down-regulation of miR-31 expression repressed aggressive phenotypes of CRC cells *in* *vitro*. (A) Inhibition of miR-31 inhibited cell proliferation of M5 and SW620 cells by CCK8 assay. (B, C) Inhibition of miR-31 reduced cell invasiveness and migration as determined by matrigel invasion chamber (B) and wound healing assays (C). Data were presented as mean ±SD. The results were reproducible in three independent experiments. * *p* < 0.05, ** *p* <0.001.

To confirm the effects of miR-31 on modulating the malignant phenotypes of CRC cells, we also investigated the change of aggressive phenotypes of CRC cells after reduced expression of miR-31. miR-31-specific inhibitor transfection was employed to inhibit miR-31 expression in M5 and SW620 cells, which had high endogenous miR-31 expression. As shown in Figure 4A, when compared with the control cells, a significantly slower proliferation rate was observed in miR-31 inhibitor-transfected cells (*p*<0.001). Moreover, matrigel invasion and wound-healing assays confirmed that the inhibition of miR-31 expression reduced the invasiveness and migration of M5 and SW620 cells, compared to the control cells (*p*<0.05, Figure 4B and 4C).

### SATB2 was one of direct targets of miR-31 directly targeted SATB2 3’UTR

**Figure 5:**
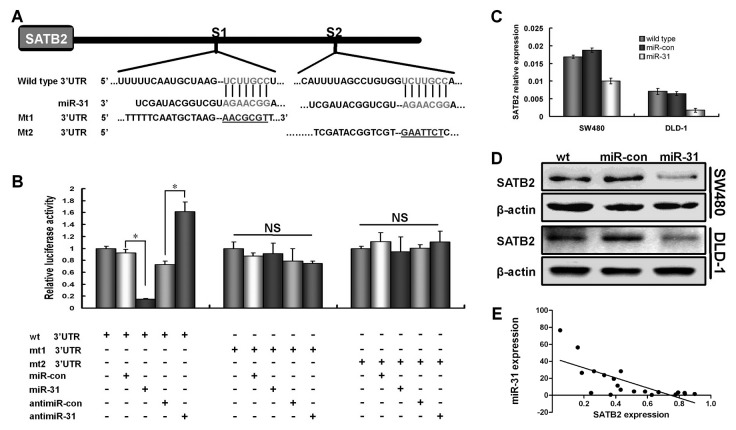
SATB2 was a direct target of miR-31 in CRC cells. (A) Schematic illustration of the predicated miR-31-binding sites (S1 and S2) in SATB2 3’-UTR. The mutant binding site was underlined. (B) Luciferase reporter assays in SW480 cells, with cotransfection of wild type or mutated 3’UTR and miRNA as indicated. NS denotes no statistical significance. (C, D) Levels of SATB2 mRNA and protein after miR-31-induced expression in CRC cell lines examined by real-time RT-PCR (C) and western blot (D). Ectopic expression of miR-31 decreased the endogenous levels of SATB2 mRNA and protein. (E) A statistically significant inverse correlation between miR-31 and SATB2 mRNA expression in CRC tissues (Spearman's correlation analysis, *r* = −0.687;  *p*=0.001, n = 20).

To search for potential targets of miR-31 that influence proliferation and migration ability of cells, we analyzed the putative miR-31 targets. The 3’UTR of SATB2 mRNA contained two complementary sites for the binding region of miR-31. We cloned a 2711 bp 3’UTR fragment containing both putative binding sites into the downstream of luciferase open reading frame of pGL3 control vector. We mutated several base pairs in the binding regions of the miR-31 binding site too (Figure 5A). An increased expression of miR-31 significantly reduced the luciferase activity of the reporter containing SATB2 3’UTR in SW480 cells, but this did not affect the luciferase activity of the empty vector control (*p*<0.001, Figure 5B). When compared with anti-miR-con transfection, an induced luciferase activity was shown in cells transfected with miR-31 inhibitor (*p*<0.001, Figure 5B). The luciferase activity of mutant reporters were unaffected by the simultaneously increased expression of miR-31 (Figure 5B), indicating that miR-31 directly bound to the 3’UTR of SATB2.

We analyzed the changes of SATB2 expression in CRC cell lines after miR-31 over-expression. We found that the over-expression of miR-31 resulted in significant reduction of SATB2 mRNA. Meanwhile, western blot assays showed that the protein levels of SATB2 were also substantially decreased after ectopic over-expression of miR-31 in SW480 and DLD-1 cells (Figure 5C and 5D). We further compared the correlation between miR-31 and SATB2 expression. We observed a good negative correlation between miR-31 and SATB2 mRNA expression (Spearman's correlation analysis, *r* = −0.687; *p*=0.001, Figure 5E). 

### The promotion of the proliferation and metastasis of CRC cells by miR-31 could be significantly attenuated by the ectopic over-expression of SATB2

**Figure 6:**
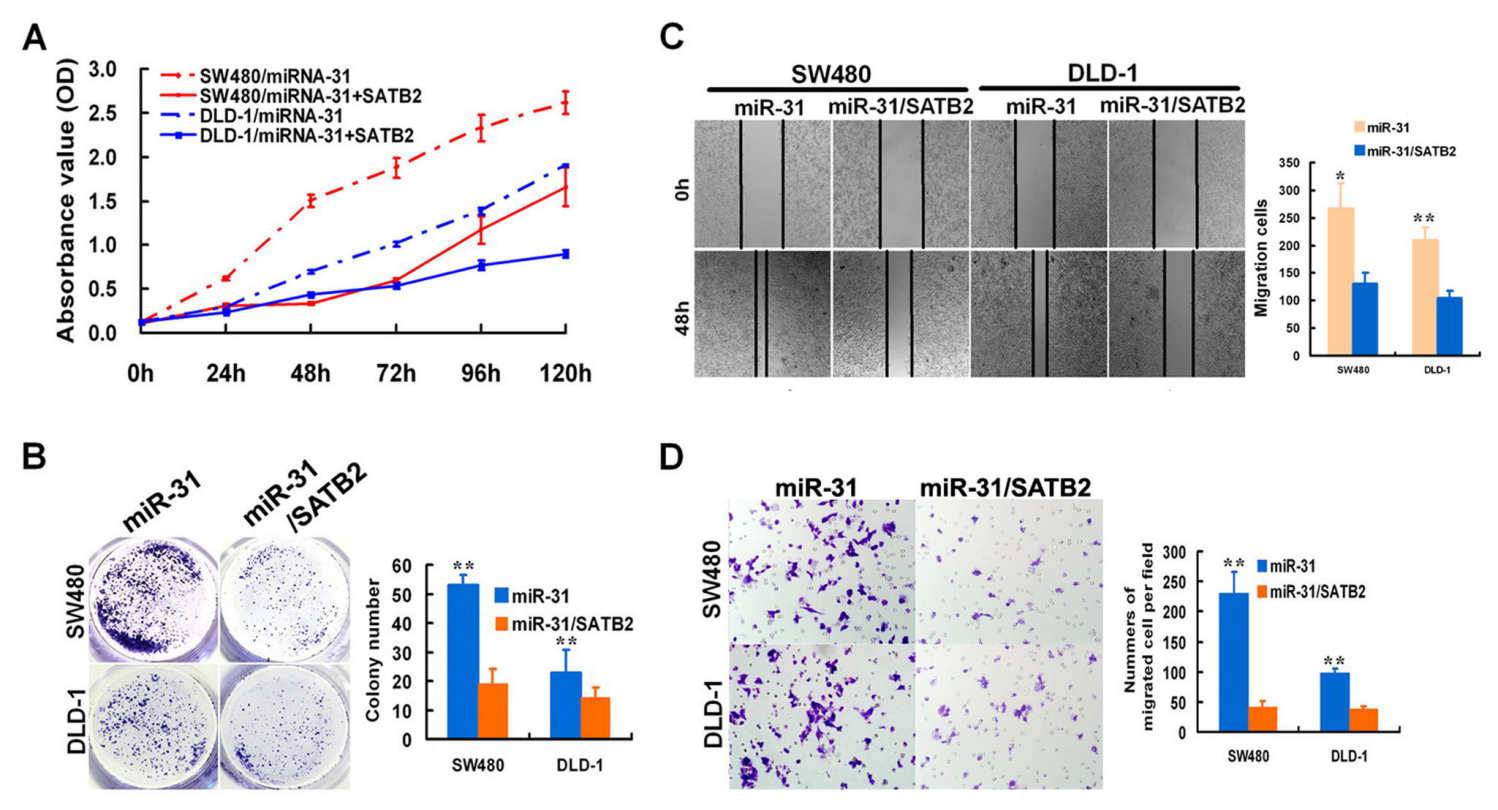
miR-31 affected cell growth, migration, and invasion by directly targeting SATB2 in CRC. Cell growth rate (A) and colony formation (B) were measured to compare the growth difference between SW480/miR-31 cells and SW480/miR-31 cells with over-expression STAB2. The over-expression of SATB2 can significantly attenuate the promotion effects of miR-31 on cell proliferation. The wound-healing (C) and matrigel invasion (D) assays were performed to investigate the migration changes of SW480/miR-31 after the ectopic expression of SATB2. SATB2 can inhibit migration and invasion of SW480/miR-31. SW480/miR-31 denoted CRC SW480 cells with stable over-expression miR-31. * *p* < 0.05, ** *p* <0.001.

To address whether the above-observed phenotype was indeed due to the suppression of SATB2, a rescue experiment was performed. SW480/miR-31 cells with stable miR-31 over-expression were transfected with pCAG-SATB2 vector which encoded the entire SATB2 coding sequence but lacked the 3’UTR. Indeed, SATB2 over-expression in SW480/miR-31 cells could inhibit both proliferation and migration ability by using CCK8, colony formation, wound-healing and matrigel invasion assays respectively, compared as that of SW480/miR-31 (*p*<0.05, Figure 6). 

### Over-expression of miR-31 induced CRC cells metastasis through EMT pathway

**Figure 7:**
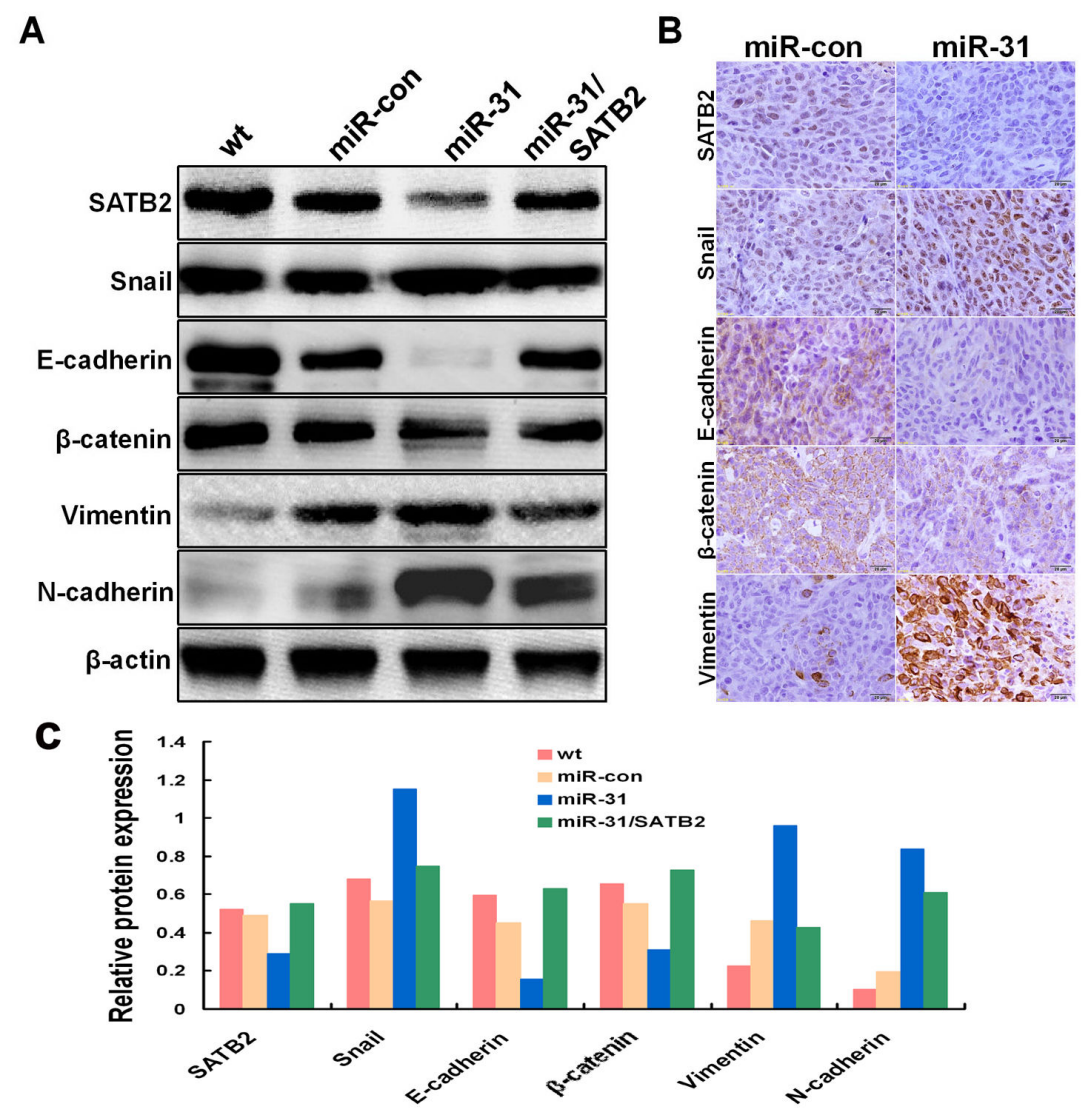
Over-expression of miR-31 induced epithelial mesenchymal transfer *in* *vitro* and *in* *vivo*. (A) Expression of epithelial markers, mesenchymal marker, Snail, and SATB2 were compared by western blot analysis in wt SW480, SW480/miR-con, SW480/miR-31 and SW480/miR-31 by transiently transfected with pCAG-SATB2 vector encoding the entire SATB2 coding sequence. β-actin was used as a loading control. (B) Immunohistochemistry staining of indicated proteins. (C) The indicated protein expression levels were quantified by comparing the gray level of each band in (A) using Quantity One Software.

We assessed the epithelial and mesenchymal markers by western blot. As expected, the expression levels of Snail and two mesenchymal makers (N-cadherin and vimentin) were strikingly up-regulated in miR-31-overexpression cells, whereas SATB2 and two epithelial marker (E-cadherin and β-catenin) levels were down-regulated. Thereafter, SATB2 over-expression could significantly attenuate the expression changes of the above markers associated with miR-31(Figure 7A).

Furthermore, IHC staining illustrated that the tumors in caecum terminus of mice originating from SW480/miR-31 cells had increased expression of Snail and vimentin. Moreover, these tumors originating from SW480/miR-31 cells had decreased expression of SATB2, E-cadherin, and β-catenin, compared with that from SW480/miR-con (Figure 7B), suggesting that over-expression of miR-31 induced the EMT of SW480 cells both *in vitro* and *in vivo*. 

## Discussion

Over the last decade, it has become evident that miRNAs are involved in the pathogenesis of several human diseases, including cancer. An increasing number of *in vitro* studies have demonstrated an important role of miR-31 in regulating tumor growth, apoptosis, metastasis and chemotherapy resistance[14,25–27]. However, miR-31 plays various functions in different cancers. The contradictory results may indicate that miR-31 is tissue specific to a significant extent. Previous studies have revealed that the miR-31 expression was positively related to advanced TNM stage[10,11] and/or deeper invasion of tumors[10] in CRC. However, less is known about the relationship between the miR-31 expression and the prognosis of patients with CRC. In our study, we found that CRC patients with higher miR-31 expression tend to have advanced T-stage, lymph node or distant metastasis, which consistent with others’ findings in CRC. Furthermore, an over-expression of miR-31 was associated with shorter overall survival of CRC patients. A multivariate Cox proportional hazard regression analysis revealed that miR-31 over-expression had a significantly worse prognostic impact on the overall survival of CRC patients independent of distant metastasis. The results indicated that, as an independent risk factor, miR-31 could serve as a prognostic marker for the survival of CRC patients.

miRNAs are well preserved in formalin-fixed tissue, making them attractive candidates for use in routinely processed tissue materials[28]. Most of the previous studies on miRNA clinical-prognosis were done by qRT-PCR using RNA extracted from human cancer tissue blocks. Cancer tissue block contains a mixture of neoplastic tumor cells and tumor related stromal cells. Using RNA extracts from whole tumors may led to erroneous results for biomarker[21]. Recent data have suggested that some miRNAs are highly expressed in stromal cells[29,30]. A major advantage of *in situ* hybridization is the ability to precisely identify positive signals at the cellular level. To date, several studies have revealed the prognostic significance of miR-31 in various carcinomas, such as breast[14], bladder[31], and lung squamous cell carcinoma[32]. To the best of our knowledge, we presented the first large-scale study that combined *in situ* hybridization to evaluate the prognostic impact of miR-31 expression in CRC.

To explore the potential biological function of miR-31 in CRC carcinogenesis and progression, we established two stable miR-31 over-expression cell lines to test the effect of miR-31 on behaviors of tumor cells. The results indicated that miR-31 performed several functions as follows: it promoted cell growth and colony formation; it induced cell cycle G1/S transition of CRC cells *in vitro*; and it incited tumorigenesis in murine model of CRC xenograft. The data were similar to the findings associated with cases of lung cancer [33]. In lung cancer cases, the knockdown of miR-31 substantially repressed lung cancer cell growth and carcinogenicity. miR-31 could activate the RAS pathway and function as an oncogene in CRC by repressing RASA1 protein[27]. Thus, the confirming results help us deducing that miR-31 is an oncogenic miRNA for CRC. In this study, we also observed that the expression level of miR-31 in patients with lymph node metastases was higher than that in patients without metastases. The expression level of miR-31 in the metastasis cells was higher than those cells with little metastatic powers. Clinicopathological analysis also found that high-level expression of miR-31 was significantly associated with lymph node metastasis and distant metastasis, which indicated that the up-regulation of miR-31 could be regarded as a predicted factor of metastasis for CRC patients. Indeed, the functional assays *in vitro* revealed that miR-31 could facilitate tumor invasion and migration. In animal models, the over-expression of miR-31 was sufficient to promote CRC cells metastasis to lung, liver, and organs in the peritoneal cavity. This association indicated that miR-31 might play a pivotal role in CRC metastasis. However, Valastyan et al. illustrated the loss of miR-31 in metastatic breast cancer cell lines and patients. They also identified that miR-31 suppressed breast cancer metastasis by a repressed cohort of pro-metastatic genes[14]. These controversial results indicated that the role of miR-31 was possibly tumor specific and highly dependent on its targets in different cancer cells. Indeed, the tissue- and time-dependent expression of miRNAs influenced protein translation during distinct cellular processes. Furthermore, the aberrant expression of their target genes affected different biological pathways with diverse function [34].

The previous studies have documented a number of deregulated miRNAs in CRC, including miR-21, miR-31 and so on. These miRNAs should exert their own functions by regulating some specific target genes. miR-21 is one of the most extensively studied miRNAs in CRC and other cancers[35–38]. It has been long believed to function as an oncogene by repressing some specific tumor suppressors such as PDCD4[39–41]. miR-31 and miR-21 can regulate their target genes as well as associated signaling pathways, and eventually enhance tumor aggressiveness. To further explore the mechanism by which miR-31 regulates tumor invasion and metastasis in CRC, we adopted some strategies that could identify miR-31 targets in CRC metastasis. Only the genes involved in metastasis cascade were considered as relevant targets with respect to the biological functions of miR-31 in CRC. We focused on SATB2, a metastasis-related gene with CRC. In our previous studies, it was found that the expression of SATB2 mRNA and protein was lower in primary CRC tissues than in their normal counterparts. Furthermore, the down-regulated expression of SATB2 is associated with metastasis and poor prognosis in CRC[19]. In addition, 3’UTR of SATB2 mRNA contains two complementary sites for the binding region of miR-31. Previous studies have identified SATB2 as a target of miR-31 in cancer-associated fibroblasts[30]. These findings suggested that SATB2 might be an important target of miR-31, wherein it regulated invasion and metastasis in CRC. Our experimental results further proved that SATB2 was a target of miR-31 in CRC cells. The activities of SATB2 3’UTR luciferase reporter were responsive to miR-31 over-expression. Endogenous SATB2 expression in both mRNA and protein decreased in miR-31 precursor-transfected CRC cells. This study clearly illustrated that miR-31 might directly regulate SATB2 expression by inducing mRNA degradation and translational suppression. Furthermore, the most important effect exerted by miR-31 on cell proliferation, invasion, and migration is partially reversed after transfection with a SATB2 expression vector. Both miR-31 and SATB2 affect migration and invasion of CRC cells but in an opposite direction. Thus, the miR-31/SATB2 pathway constitutes a previously unrecognized carcinogenesis and progression regulator of CRC. 

 In addition, we found that the reintroduction of miR-31 into SW480 cells induced epithelial-mesenchymal transition (EMT), indicating the down-expression of epithelial markers and up-expression of the mesenchymal markers. However, it did not exhibit the direct evidence of EMT-related molecules that were regulated by miR-31/SATB2 in our studies. SATB2 is one member of nuclear matrix-attachment proteins family that recognizes AT-rich DNA sequences at the base of looped-out chromatin. SATB2 are involved in various important genetic processes, such as chromatin condensation, interaction with other chromatin remodeling complexes, and regulation of transcription[42–44]. SATB2 is involved in global chromatin remodeling. Furthermore, it may be responsible for the major changes observed in global gene expression. So, we put forward a hypothesis that SATB2 might remodel chromatin and further regulate the expression of Snail, a key factor in EMT, which needs to be confirmed in further studies. 

In summary, we observed that miR-31 expression was up-regulated in CRC, and such over-expression was significantly associated with aggressiveness of CRC patients, indicating poor clinical outcome. Our results suggested that the up-regulation of miR-31 played an important role in CRC cell proliferation, invasion, and metastasis *in vitro* and *in vivo* through direct repressing SATB2, proving the miR-31 effect on tumorigenesis and progression. Hence, miR-31 could be considered as a novel therapeutic target for patients with CRC. 

## Supporting Information

Table S1 **Primer sequences for detection and miRNA sequences.**
(XLS)
